# Differential expressions of PD-1, PD-L1 and PD-L2 between primary and metastatic sites in renal cell carcinoma

**DOI:** 10.1186/s12885-019-5578-4

**Published:** 2019-04-16

**Authors:** Xingming Zhang, Xiaoxue Yin, Haoran Zhang, Guangxi Sun, Yaojing Yang, Junru Chen, Xudong Zhu, Peng Zhao, Jinge Zhao, Jiandong Liu, Ni Chen, Jia Wang, Pengfei Shen, Hao Zeng

**Affiliations:** 1 Department of Urology, West China Hospital, Sichuan University, Chengdu, People’s Republic of China 610041; 2 Institute of Urology, West China Hospital, Sichuan University, Chengdu, People’s Republic of China 610041; 3 Department of Pathology, West China Hospital, Sichuan University, Chengdu, People’s Republic of China 610041; 4 Department of Urology, Institute of Urology, West China Hospital, Sichuan University, No. 37 Guoxue Xiang, Chengdu, Sichuan People’s Republic of China 610041

**Keywords:** Renal cell carcinoma, Primary tumor, Metastases, Immunological checkpoint, Differential expression

## Abstract

**Background:**

In clinical practice, the detection of biomarkers is mostly based on primary tumors for its convenience in acquisition. However, immune checkpoints may express differently between primary and metastatic tumor. Therefore, we aimed to compare the differential expressions of PD-1, PD-L1 and PD-L2 between the primary and metastatic sites of renal cell carcinoma (RCC).

**Methods:**

Patients diagnosed with RCC by resection or fine needle aspiration of metastasis were included. Immunohistochemistry (IHC) was applied to detect PD-1, PD-L1 and PD-L2 expressions. SPSS 22.0 was applied to conduct Chi-square, consistency tests and Cox’s proportional hazards regression models. GraphPad Prism 6 was used to plot survival curves and R software was used to calculate Predictive accuracy (PA).

**Results:**

In the whole cohort (*N* = 163), IHC results suggested a higher detection rate of PD-L1 in the metastasis than that of the primary site (χ2 = 4.66, *p* = 0.03), with a low consistent rate of 32.5%. Among different metastatic tumors, PD-1 was highly expressed in the lung/lymph node (65.3%) and poorly expressed in the brain (10.5%) and visceral metastases (12.5%). PD-L1 was highly expressed in lung/lymph node (37.5%) and the bone metastases (12.2%) on the contrary. In terms of survival analysis, patients with PD-1 expression either in the primary or metastasis had a shorter overall survival (OS) (HR: 1.59, 95% CI 1.08–2.36, *p* = 0.02). Also, PD-L1 expression in the primary was associated with a shorter OS (HR 2.55, 95% CI 1.06–6.15, *p* = 0.04). In the multivariate analysis, the predictive accuracy of the whole model for PFS was increased from 0.683 to 0.699 after adding PD-1.

**Conclusion:**

PD-1, PD-L1 and PD-L2 were differentially expressed between primary and metastatic tumors. Histopathological examination of these immune check points in metastatic lesions of mRCC should be noticed, and its accurate diagnosis may be one of the effective ways to realize the individualized treatment.

**Electronic supplementary material:**

The online version of this article (10.1186/s12885-019-5578-4) contains supplementary material, which is available to authorized users.

## Background

Renal cell carcinoma (RCC) is originated from the epithelium of renal tubules. Clear cell RCC (ccRCC) is the most common type of RCC (82–90%) [1, 2]. Among all RCC patients, nearly 20–30% of them were initially diagnosed as metastatic RCC (mRCC), and 20–40% of patients with localized disease will eventually develop metastatic disease after surgery [3]. RCC is not sensitive to radiotherapy and chemotherapy, and the efficiency of cytokines was limited, especially accompanied by significant adverse events [4–6]. Although the development of targeted agents, such as tyrosine kinase inhibitor (TKI) and the drug targeting the mammalian target of rapamycin (mTOR), have dramatically improved the patient’s survival time in the past two decades, mRCC patients still failed to receive satisfactory outcomes [7].

To achieve better outcomes of patients with mRCC, immune check point inhibitors have been successfully introduced into clinical practice. The key procedure of anti-tumor respond is the activation of T cell and tumor cells can inhibit this process through binding its immune checkpoints to receptors expressed on T-cell, thus leading to immune escape. Development of immune checkpoints inhibitors (PD-1 and PD-L1 antibodies) have been approved by the U.S. FDA as the standard second-line treatment for mRCC and even in the first-line for moderate to high risk of mRCC [8, 9]. In addition to RCC, the expression of PD-1/PD-L1 in solid tumors such as melanoma, bladder cancer, hematological malignancies, liver cancer, and non-small cell lung cancer can be used as prognostic markers to predict therapeutic effect of checkpoints inhibitors (CPIs) [10–20]. Although over-expression of PD-L1 in renal cell carcinoma has been shown to be associated with poor pathological features and prognosis [21, 22] its expression in primary tumors failed to predict whether patients can benefit from inhibiting PD-1/PD-L1 axis [11, 13]. In fact, 18% of RCC patients with PD-L1 negative also benefited from CPIs treatment [11], and there were still a large proportion of PD-L1 positive patients did not response to the treatment [16]. Explanations to the discrepancy between the trial results and expectation involve many possibilities. Among them, the heterogeneity of the tumor may play an important role, especially the heterogeneity between the primary and metastatic tumors.

In clinical practice, the detection of biomarkers is mostly based on primary tumors due to its convenience in acquisition. However, the tumor microenvironment may have changed at the time of disease progression and treatment administration (e.g., CPIs). Therefore, the value of assessing the primary tumor has been challenged for its poverty in providing full detailed or more accurate biological information in guiding treatment by targeted agents or CPIs.

Therefore, we firstly assessed and analyzed the differential expressions of several key immune checkpoints (PD-1, PD-L1/2) between the primary and metastatic tumors of RCC in Asian patients, and also aimed to analyze the relationship between the differential expressions and clinicopathologic parameters, then to estimate its significance in predicting patient’s survival outcomes.

## Methods

### Patients and samples

We included patients diagnosed with mRCC in West China Hospital, Sichuan University from 2009.1 to 2016.11. Inclusion and exclusion criteria were described as in our previous study [23]. This study was approved by the Ethics Committee of West China Hospital, Sichuan University and conformed to the ethical guidelines of the 1975 Declaration of Helsinki. The pathological diagnosis was retrieved and confirmed by two experienced genitourinary pathologists independently (Ni Chen and Xiaoxue Yin). Clinical and pathological data were recorded in detail. The clinical outcomes included PFS and OS, and the definition of PFS and OS was described as in our previous study [23]. At the cut-off point, the median follow-up time was about 36 months (16–70 months).

Immunohistochemistry (IHC) was applied to detect PD-1, PD-L1 and PD-L2 expressions. IHC staining was performed by using anti-PD-1 monoclonal antibody (Abcam, clone number: ab52587), anti-PD-L1 monoclonal antibody (Zhongshan Golden Bridge, clone number: ZM-0170) and anti-PD-L2 monoclonal antibody (Abcam, clone number: ab200377) at a 1:200 dilution, respectively. Positive signal of PD-1, PD-L1 and PD-L2 were on the cell membrane. We evaluated staining intensities based on a scale as previously described [23]. The results were recorded as the following categories: staining intensity of null (0), weak (1+), moderate (2+) and strong (3+). Positivity was defined as the positive signal detected on > 5% tumor-infiltrating T cells (PD-1) or tumor cells (PD-L1 and PD-L2) with staining intensity ≥1+. Two experienced genitourinary pathologists (Ni Chen and Xiaoxue Yin) independently assessed all IHC staining results.

### Data analysis

We calculated mean and SD for continuous parameters, and proportions for categorical parameters. Chi-square and non-parametric test, conducted by SPSS 22.0, were applied to analyze the statistical difference. For the analysis of the consistency of expression between the primary and metastatic tumors, Kappa test was adopted for the evaluation by conducting SPSS 22.0, and the agreement was classified into the following four levels: 1) slight agreement: Kappa≤0.2; 2) fair agreement: 0.4 < Kappa≤0.6; 3) substantial agreement: 0.6 < Kappa≤0.8; 4) almost perfect agreement: Kappa > 0.8. For the analysis of PFS and OS, we used GraphPad Prism 6 to plot Kaplan-Meier survival curves and SPSS 22.0 to conduct Cox’s proportional hazards regression models. In addition, R software was used to calculate Predictive accuracy (PA). *P* < 0.05 was considered significant in all results.

## Results

### Baseline data

From January, 2009 to November, 2016, A total of 163 patients from West China Hospital, Sichuan University were included (107 male, 56 female), with an average age of 53.9 years. Eighty-three of them were paired (with both primary and metastatic samples), and the other 80 were only metastatic specimen (Additional file 1: Table S1).

### Expressions of PD-1, PD-L1 and PD-L2 in the whole and paired cohort

**Fig. 1:**
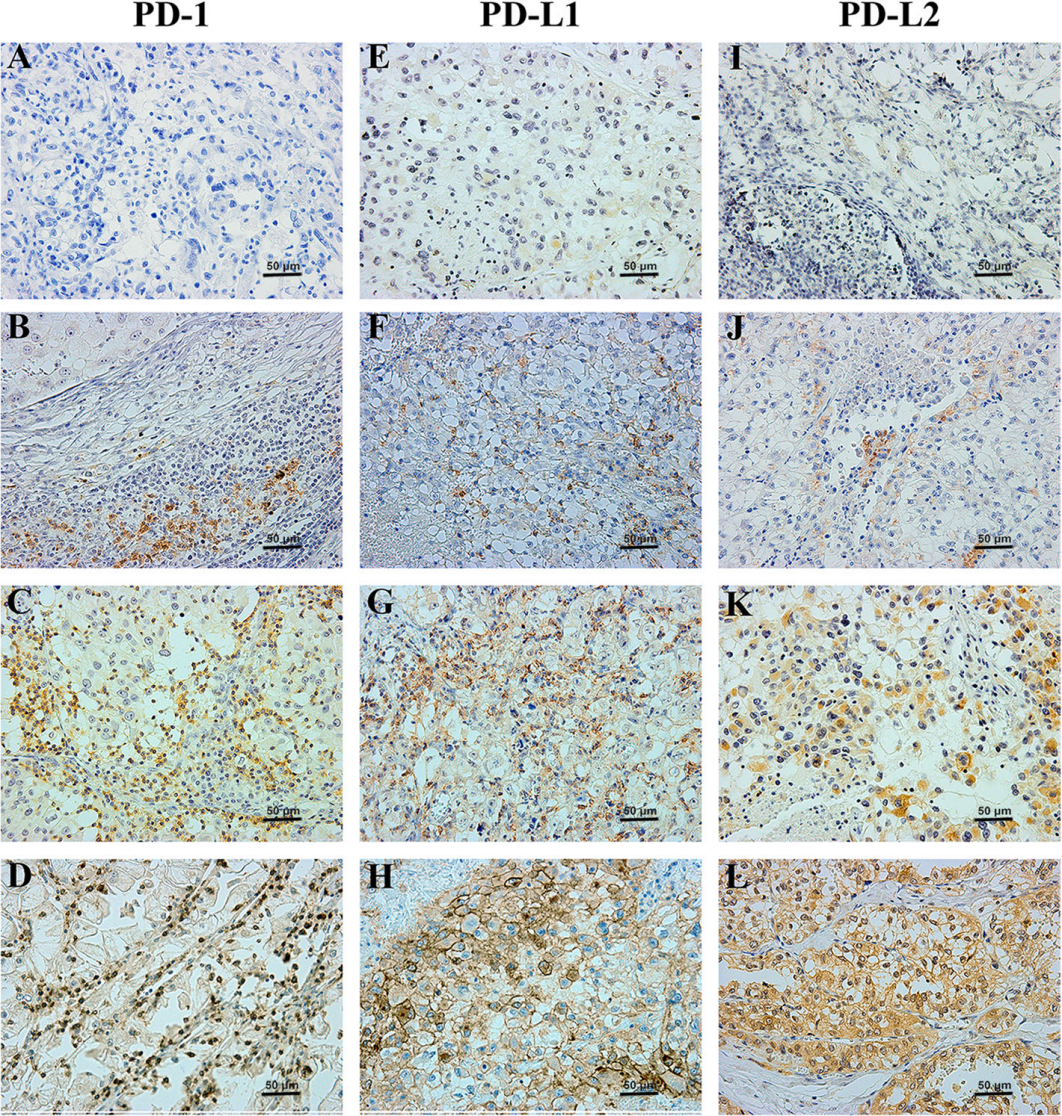
Immunohistochemical staining against PD-1, PD-L1 and PD-L2. Representative images of FFPE samples immunostained with PD-1 (**a-d**), PD-L1 (**e-h**) and (**i-l**), original magnification: 200×. A, E and I represented negative expression of PD-1, PD-L1 and PD-L2, respectively. B-D, F-H and J-L represented different positive intensities (1+, 2+ and 3+) of PD-1, PD-L1 and PD-L2, respectively

Figure 1a showed negative expression of PD-1 in RCC tumor. In the whole cohort (*N* = 163), PD-1 was detected in 76 patients (46.6%). PD-1 was mainly expressed on the membrane of tumor infiltrating immune cells (Fig. 1b-d). Figure 1e and i showed negative expressions of PD-L1 and PD-L2 in RCC tumors. PD-L1 and PD-L2 were detected in 53 patients (32.5%) and 43 patients (26.4%), respectively. Both PD-L1 and PD-L2 were mainly expressed on the surface of tumor cell membrane (Fig. 1f-h, Fig. 1j-l).

The associations between clinical pathological parameters and expressions in the primary or metastatic tumors of the three immune check points were shown in Table 1. PD-1 positive was associated with metastatic sites of lymph node(*p* < 0.001), brain(*p* < 0.001) and viscera(*p* = 0.02. Expression of PD-L1 was correlated with metastatic sites of lymph node(*p* = 0.02) and bone(*p* = 0.01). However, PD-L2 was only associated with nephrectomy status(*p* = 0.02).

**Table 1:** Relationship between the expressions of PD-1, PD-L1 and PD-L2 and clinicopathological parameters in the whole cohort

	PD-1		*P* Value	PD-L1		*P* Value	PD-L2		*P* Vaule
	Positive	Negative		Positive	Negative		Positive	Negative	
Total	76(46.6)	87(53.4)		53(32.5)	110(67.5)		43(26.4)	120(73.6)	
Age, *n* (%)			0.65			0.27			0.35
< 50y	27(49.1)	28(50.9)		21(38.2)	34(61.8)		17(30.9)	38(69.1)	
≥ 50y	49(45.4)	59(54.6)		32(29.6)	76(70.4)		26(24.1)	82(75.9)	
Gender, *n* (%)			0.97			0.18			0.16
Male	50(46.7)	57(53.3)		31(29.0)	76(71.0)		32(29.9)	75(70.1)	
Female	26(46.4)	30(53.6)		22(39.3)	34(60.7)		11(19.6)	45(80.4)	
ISUP, *n* (%)			0.13			0.81			0.57
< 3	12(34.3)	23(65.7)		9(25.7)	26(74.3)		7(20.0)	28(80.0)	
≥ 3	55(50.0)	55(50.0)		40(36.4)	70(63.6)		30(27.3)	80(72.7)	
Histological Type, *n*(%)			0.22			0.78			0.52
ccRCC	56(44.1)	71(55.9)		42(33.1)	85(66.9)		32(25.2)	95(74.8)	
Non-ccRCC	20(55.6)	16(44.4)		11(30.6)	25(69.4)		11(30.6)	25(69.4)	
Pathology, *n* (%)									
Sarcoma	7(87.5)	1(12.5)	0.07	4(50.0)	4(50.0)	0.66	3(37.5)	5(62.5)	0.69
Necrosis	19(63.3)	11(36.7)	0.59	11(36.7)	19(63.3)	0.33	6(20.0)	24(80.0)	0.09
Nephrectomy			0.22			0.18			0.02
Yes	68(49.6)	69(50.4)		48(35.0)	89(65.0)		39(28.5)	98(71.5)	
No	7(35.0)	13(65.0)		4(20.0)	16(80.0)		1(5.0)	19(95.0)	
ECOG, *n* (%)			0.77			0.15			0.62
0–1	53(47.3)	59(52.7)		42(37.5)	70(62.5)		29(25.9)	83(74.1)	
≥ 2	20(50.0)	20(50.0)		10(25.0)	30(75.0)		12(30.0)	28(70.0)	
IMDC, *n* (%)			0.11			0.26			0.83
Low	10(30.3)	23(69.7)		9(27.3)	24(72.7)		9(27.3)	24(72.7)	
Intermediate	41(51.3)	39(48.8)		29(36.3)	51(63.7)		19(23.8)	61(76.2)	
High	15(51.7)	14(48.3)		6(20.7)	23(79.3)		6(20.7)	23(79.3)	
T stage, *n* (%)			0.691			0.09			0.91
< 3	43(58.9)	30(41.1)		31(42.5)	42(57.5)		20(27.4)	53(72.6)	
≥ 3	14(43.8)	18(56.2)		5(22.7)	17(77.3)		6(27.3)	16(72.7)	
Metastasis, *n* (%)									
Lung	13(54.2)	11(45.8)	0.46	11(45.8)	13(54.2)	0.21	7(29.2)	17(70.8)	0.91
Lymph node	37(77.1)	11(22.9)	< 0.001	23(47.9)	25(52.1)	0.02	18(37.5)	30(62.5)	0.09
Bone	18(43.9)	23(56.1)	0.64	6(14.6)	35(85.4)	0.002	10(24.4)	31(75.6)	0.54
Brain	2(10.5)	17(89.5)	0.001	7(36.8)	12(63.2)	0.82	2(10.5)	17(89.5)	0.07
Adrenal	1(16.7)	5(83.3)	0.13	1(16.7)	5(83.3)	0.35	3(50.0)	3(50.0)	0.23
Viscera	3(18.8)	13(81.3)	0.02	5(31.3)	11(68.8)	0.78	6(37.5)	10(62.5)	0.38
Others	8(40.0)	12(60.0)	0.49	7(35.0)	13(65.0)	0.96	3(15.0)	17(85.0)	0.16
Treatment, *n* (%)									
Cytokine	11(50.0)	11(50.0)	0.76	4(18.2)	18(81.8)	0.09	8(36.4)	14(63.6)	0.23
Targeted therapy	32(50.0)	32(50.0)	0.49	24(37.5)	40(62.5)	0.54	15(23.4)	49(76.6)	0.83
Radiotherapy	7(50.0)	7(50.0)	0.81	4(28.6)	10(71.4)	0.69	3(21.4)	11(78.6)	0.79
Chemotherapy	4(50.0)	4(50.0)	0.89	4(50.0)	4(50.0)	0.34	3(37.5)	5(62.5)	0.39

*Abbreviations*: *ISUP* International Society of Urological Pathology, *ccRCC* clear cell renal cell carcinoma, *ECOG* Eastern Cooperative Oncology Group, *IMDC* International Metastatic Renal-Cell Carcinoma Database Consortium

After stratified patients’ specimens into primary tumors and specific metastatic sites, as shown in Table 2, PD-1 was positive in 31.3%(26/83) of primary tumors and 42.0%(73/173) of metastatic tumors (*p* = 0.10). However, PD-1 expression differences between the primary and metastatic sites of lung/lymph node (65.3%, *P* < 0.001), brain (10.5%, *P* < 0.001) and viscera (12.5%, *p* = 0.01) were found to be statistically significant. For PD-L1, the expression rates were 24.1%(20/83) in primary tumors and 28.7%(50/174) in metastasis (*p* = 0.44). Yet, the expression differences between the primary and metastatic sites were also observed in lung/lymph node (37.5%, *p* = 0.03) and bone (12.2%, *p* = 0.01). When comes to PD-L2, there were no statistical expression differences between the primary (16.9%) and metastasis (23.6%) or any metastatic sites.

**Table 2:** Differential expressions of PD-1,PD-L1 and PD-L2 between the primary and metastatic tumors

	Primary(*N* = 83)	Metastasis (*N* = 163)^a^						
	*n*(%)	Lung/lymph node *n* = 72	Bone *n* = 41	Brain *n* = 19	Viscera *n* = 16	Adrenal gland *n* = 6	Others *n* = 20	Total *n*(%)
PD-1								
Negative	57(68.7)	25(34.7)	28(68.3)	17(89.5)	14(87.5)	5(83.3)	12(60.0)	101(58.0)
Positive	26(31.3)	47(65.3)	13(31.7)	2(10.5)	2(12.5)	1(16.7)	8(40.0)	73(42.0)
*P* value		< 0.001	0.77	< 0.001	0.01	0.21	0.85	0.11
PD-L1								
Negative	63(75.9)	45(62.5)	36(87.8)	13(68.4)	11(68.8)	5(83.3)	14(70.0)	124(71.3)
Positive	20(24.1)	27(37.5)	5(12.2)	6(31.6)	5(31.3)	1(16.7)	6(30.0)	50(28.7)
*P* value		0.03	0.01	0.77		0.51	0.89	0.44
PD-L2								
Negative	69(83.1)	55(76.4)	31(75.6)	17(89.5)	10(62.5)	3(50.0)	17(85.0)	133(76.4)
Positive	14(16.9)	17(23.6)	10(24.4)	2(10.5)	6(37.5)	3(50.0)	3(15.0)	41(23.6)
*P* value		0.99	0.21	0.16	0.17	0.12	0.34	0.22

^a^11 cases have two metastatic sites

In the paired cohort, the concordance rate of PD-1 expression between the primary and metastasis was 57.8% (48/83, Kappa = 0.168, *p* = 0.09), with no significant expression difference between the primary and metastasis (χ^2^ = 2.795, *p* = 0.09). Meanwhile, the concordance rate of PD-L1 expression was only 32.5% (27/83, Kappa = 0.229, *p* = 0.03), with a significant expression difference between the primary and metastasis (χ2 = 4.664, *p* = 0.03). For PD-L2, the concordance rate was 73.5% (61/83, Kappa = 0.193, *p* = 0.07), with no statistical expression difference between the primary and metastasis (χ2 = 3.241, *p* = 0.07). We further stratified patients into specific metastatic sites, as shown in Additional file 2: Table S2, the significance was unavailable for several metastatic sites due to the limited quantities. However, we could find that PD-1 was differentially expressed between the primary tumor and brain metastasis(χ2 = 7, *p* = 0.01). The detection rate of PD-L1 was numerically higher in the metastasis of lung/lymph node than that of the primary tumor (38.8% vs. 24.1%), with a borderline statistical significance (χ2 = 3.056, *p* = 0.08). For PD-L2, the detection rate of bone was higher than that of the primary tumor (χ2 = 4.5, *p* = 0.03).

### Relationship between differential expressions of PD-1, PD-L1&2 and clinical pathological parameters

As shown in Additional file 3: Table S3, the differential expression of PD-1 was only correlated with gender. Compared to female patients (37.9%, 11/29), a higher concordance rate of PD-1 was observed in male patients (68.5%, 37/54). In patients with brain metastasis, the concordance rate was 100%, which was significantly different from those with non-brain metastasis(*p* = 0.02). As for PD-L1, the concordance rate was 100% in patients treated with cytokine therapy, which was predominantly higher than those who didn’t received the treatment. However, no factor was associated with the differential expression of PD-L2.

### Associations of PD-1, PD-L1 and PD-L2 expressions with clinical outcomes

Until follow-up date, 156 of patients (95.7%) showed progression, 95 of patients (58.3%) were dead. The median PFS was 23.0 months (IQR:9.0–46.0), and the median OS was 36.0 month (IQR:16.0–70.0). In 64 patients with TKI therapy, the median PFS and OS were 21.0 and 52.0 months, respectively.

**Fig. 2:**
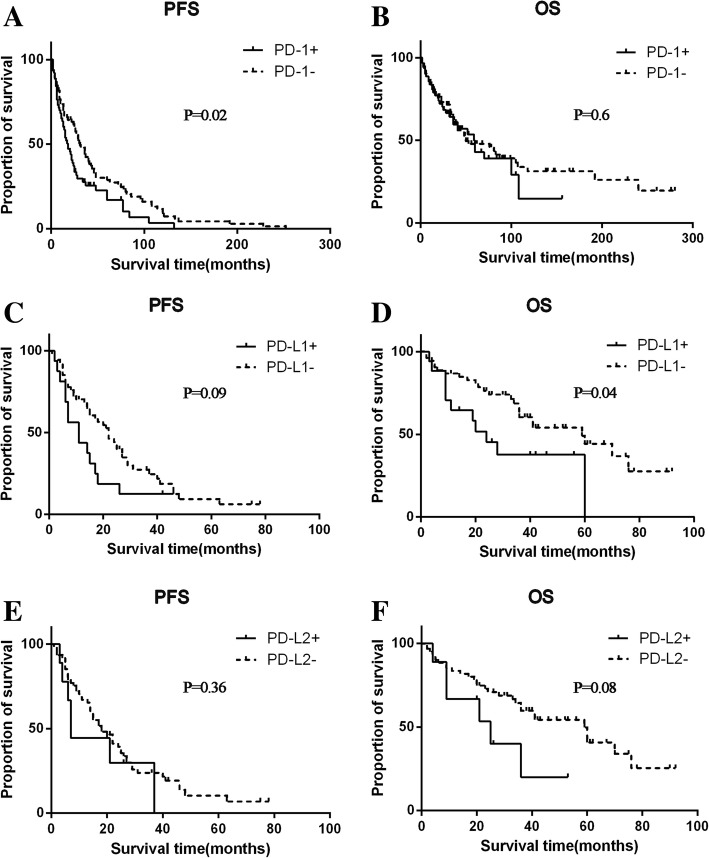
The association of expressions of immune checkpoints with clinical outcomes in patients with RCC. Representative images of kaplan–Meier survival curves of PD-1 expression either in the primary or metastatic tumors in the whole cohort (**a-b**), PD-L1 expression in the primary tumor (**c-d**) and PD-L2 expression in the primary tumor (**e-f**)

In the whole cohort (*N* = 163), PD-1 expression either in the primary or metastatic tumor was correlated with PFS (HR 1.59, 95%CI 1.08–2.36, *p* = 0.02, Fig. 2a), and no statistical significance was found for OS (Fig. 2b). However, no significant association was found between PD-L1 and PD-L2 expression and clinical outcomes. In all metastatic tumors, expressions of PD-1, PD-L1 and PD-L2 were not associated with PFS and OS. In the paired patients (*N* = 83), PD-L1 expression in primary tumor was numerically associated with PFS (Fig. 2c) and statistically associated with OS (HR 2.55, 95%CI 1.06–6.15, *p* = 0.04, Fig. 2d). However, its expression in metastatic tumors was not associated with PFS nor OS. PD-1 and PD-L2 had no impact on both PFS and OS in the paired patients.

### Multivariate analysis of PFS and OS in the whole and paired cohort

In whole cohort, univariate analysis demonstrated that ISUP≥3, nephrectomy, ECOG score, IMDC classification, metastatic status, serum WBC and PD-1 positive either in the primary or metastatic tumor were correlated with PFS (Additional file 4: Table S4). Further multivariate analysis showed that IMDC was an independently predictive factor for PFS, with a predictive accuracy (PA) of 0.683 in whole cohort. After adding expression of PD-1 either in the primary or metastatic tumor into the Cox regression model, the PA was increased to 0.699. In terms of OS, time interval from diagnosis to metastasis was an independent predictive factor (*P* = 0.02) with PA of 0.747(Table 3).

**Table 3:** Multivariate analysis of PFS and OS in all patients (*N* = 163)

	Cox’s regression for PFS				Cox’s regression for OS			
	HR	95% Cl	*P* value	PA	HR	95% Cl	*P* value	PA
Gender								
Male vs female	1.74	0.82–3.69	0.15	0.535	1.31	0.26–6.44	0.75	0.548
ISUP								
≥ 3 vs < 3	2.21	0.89–5.47	0.09	0.568	12.8	0.72–227.37	0.08	0.595
Nephrectomy								
Yes vs No	0.46	0.12–1.67	0.24	0.561	0.22	0.03–1.73	0.15	0.578
ECOG score								
≥ 2 vs 1	0.73	0.28–1.91	0.51	0.580	1.62	0.11–25.78	0.73	0.572
IMDC				0.656				0.647
Low	Ref.	Ref.	0.04		Ref.	Ref.	0.87	
Intermediate	1.15	0.48–2.78	0.76		1.08	0.11–10.29	0.95	
High	3.98	1.14–13.92	0.03		0.55	0.02–14.58	0.72	
Synchronic metastasis								
Yes vs No	0.99	0.51–1.89	0.97	0.618	65.6	2.28–1888.34	0.02	0.674
BMI					1.47	0.98–2.21	0.06	0.615
Laboratory parameters								
WBC	1.01	0.99–1.02	0.35	0.513	1.003	0.97–1.04	0.87	0.610
PD-1 positive (P or M)^a^	1.27	0.62–2.61	0.51	0.554				
Cox model without PD-1				0.683				0.747
Cox model with PD-1				0.699				

^a^P or M, primary or metastatic tumor

*Abbreviations*: *ISUP* International Society of Urological Pathology, *ECOG* Eastern Cooperative Oncology Group, *IMDC* International Metastatic Renal-Cell Carcinoma Database Consortium, *BMI* Body Mass Index

In paired patients, univariate analysis showed that IMDC was an influencing factor for PFS. IMDC, interval time from diagnosis to metastasis interval, cytokine therapy, HGB, ALP, LDH and serum Na^+^ level were influencing factors for OS (Additional file 5: Table S5). Expressions of PD-1, PD-L1 and PD-L2 in primary or metastatic sites showed no predictive value for PFS. As for OS, PD-L1 was a risk factor (*P* = 0.04), however, other checkpoints showed no predictive value. Subsequent multivariate analysis suggested that IMDC was an independently risk factor for PFS in the paired patients, with a PA of this model as 0.676. As for OS, nephrectomy and WBC were independently influencing factors, and the PA of OS model was 0.754. After adding PD-L1 expression in primary tumor, the PA was increased to 0.757 (Table 4).

**Table 4:** Multivariate analysis of PFS and OS in paired patients (*N* = 83)

	Cox’s regression for PFS				Cox’s regression for OS			
	HR	95% Cl	*P* value	PA	HR	95% Cl	*P* value	PA
ISUP								
≥ 3 vs < 3	2.18	0.89–5.31	0.09	0.546	12.1	0.99–147.1	0.05	0.576
Nephrectomy								
Yes vs No					0.03	0.004–0.25	0.001	0.525
IMDC				0.615				0.618
Low	Ref.	Ref.	0.04		Ref.	Ref.	0.17	
Intermediate	1.12	0.5–2.51	0.78		1.21	0.35–4.18	0.8	
High	2.54	1.08–5.97	0.03		2.71	0.76–9.58	0.12	
Laboratory parameters								
WBC					1.02	1.01–1.04	0.002	0.624
Na					0.93	0.82–1.06	0.26	0.556
PD-L1 positive (P)^a^					0.98	0.35–2.77	0.97	0.583
Cox model without PD-L1								0.754
Cox model with PD-L1								0.757

^a^P, Primary tumor

*Abbreviations*: *ISUP* International Society of Urological Pathology, *IMDC* International Metastatic Renal-Cell Carcinoma Database Consortium

## Discussion

In the present study, we firstly compared expressions of several checkpoints (PD-1, PD-L1/2) in primary RCC and their metastases among Asian population. We also evaluated the correlations between clinicopathological parameters and discordant expressions of PD-1, PD-L1 and PD-L2. Furthermore, the value of expression in metastases of these immune checkpoints in predicting patient’s prognosis was assessed.

It has been demonstrated that PD-L1 expression was positively associated with efficacy of immune checkpoint inhibitors among malignant tumors [24–26], however, the situation was not the same in RCC [11]. Because of its inherent heterogeneity, tumors of RCC were potentially characterized as discrepant in expressions of immune checkpoints among different sites of tumors, especially the primary and metastases. Several studies have shown that gene expression profiles and biomarkers were differentially expressed between the primary and metastatic tumors, such as breast cancer, gastric cancer, etc. [27–30]. Similarly, an identical phenomenon was observed in RCC [31–33]. The differential expression suggested that the evaluation of PD-L1 expression in metastasis might give a more accurate prediction of curative effect of PD-1/PD-L1 inhibitors [34, 35]. Although Callea et al. had analyzed the expression of PD-L1 in 56 patients with matched primary and metastatic tumors, they reached the conclusion that PD-L1 was non-differentially expressed between primary and metastatic tumors. Anyway, they did found that PD-L1 was differentially expressed in the same tumor—the higher the nuclear grade was, the more evident expression of PD-L1 was observed, suggesting that PD-L1 expression was possibly required to be evaluated in metastases in order to more accurately predict the therapeutic effect of immune checkpoint inhibitors [35]. However, the study included a limited number of cases and only analyzed the expression of PD-L1. In another study, Giraldo et al. enrolled 135 primary RCC tumors and 51 lung metastases derived from RCC to analyze the differential expressions of PD-1, PD-L1, PD-L2 and LAG-3. Their results showed that when PD-1 was combined with PD-L1 or PD-L2, the expression of PD-1 in metastasis, but not the primary, was an independent risk factor for OS, suggesting that the differential expressions of immune checkpoints between the primary and metastatic tumors were correlated with patients’ prognosis [36]. Since this study only included a limited number of lung metastasis and these metastases were not matched with the primary tumors, the differential expressions of immune checkpoints between the primary and metastatic tumors still need to be further verified.

Until now, studies reporting on differential expressions of immune checkpoints in RCC were in shortage, especially no study reported data of Asian patients. Immune check points might differentially expressed within an individual or among individuals for tumor heterogeneity. Therefore, we firstly detected and compared the differential expressions of several immune checkpoints (PD-1, PD-1/2 and TIM-3) between primary and metastatic tumors in Chinese RCC patients, hopefully, to provide a laboratory basis for predicting the efficacy of immunotherapy in patents with mRCC and ultimately offer some insights into the realization of individualized treatment. Therefore, based on results of the present study, we concluded that the expression differences of PD-1, PD-L1&2 between primary and metastatic tumor were significant, which was different from the previous report of Western population [35]. The underlying reason for the difference was not clear. However, it might be attributed to the regional difference. Regional or racial difference could be one of the multi-factors in contributing to heterogeneity and it has also been proved that these differences have evident influence on the incidence, risk and prognosis of RCC [37]. What’s more, the population included in our study had a lower rate of T stage≥3 than that of Callea et al. reported. In addition to this, the detection rates of PD-1, PD-L1 and PD-L2 were different from other studies. In the present study, the overall detection rates were 46.6% (PD-1), 32.5% (PD-L1) and 26.4% (PD-L2), respectively. Notably, the detection rates of these checkpoints in the primary tumor were 31.1% (PD-1), 24.1% (PD-L1), 16.9% (PD-L2), respectively. And the reported detection rates of PD-1, PD-L1 and PD-L2 in the primary tumor were 56.6% [38], 13.0–66.3% [21, 22, 39, 40], and 21.0% [41] respectively. Besides, in our study, the detection rates of PD-1, PD-L1 and PD-L2 in the metastasis were 42.0, 28.7 and 23.6% respectively, while the reported data of other studies were 25.5% [22], 10.0–75.0% [36, 42] and 29.0% [36], respectively. Except for racial or regional differences, several other concerned factors might contribute to the above differences could be different detection method, efficiency of the primary antibody and evaluation criterion of positivity. Another noteworthy point was that, it has been reported that PD-1 was related to T stage, nuclear grade, sarcomatous differentiation and necrosis, and PD-L1 expression was related to T stage, nuclear grade, necrosis and ECOG [21, 39, 43]. The present study failed to reach similar results, which might be reason of small number of included patients and disease status (metastatic or advanced RCC).

In the present study, PD-1, PD-L1, and PD-L2 were differentially expressed between the primary and metastatic tumors. All of them had a higher expression rate in metastasis than that of the primary tumor. These checkpoints had poor concordance rate between the primary and metastatic tumors, suggesting that the assessment of primary tumor was insufficient to accurately predict patient’s treatment outcomes and prognosis. What’s more, PD-1, PD-L1, and PD-L2 had differential expressions in various metastasis. Therefore, the treatment outcomes might be correlated with different metastatic sites. Since both PD-1 and PD-L1 had higher expression rate in lung/lymph node, it seems that patients with metastasis in lung and lymph node were potential targets for immune therapy.

Limitations of the present study were: PD-L1/2 expressions were: 1) not assessed in tumor infiltrating immune cells; 2) sample size was limited, especially the number of paired patients; 3) different types of specimens of tumors might have influences on the results of IHC. However, we still have reached several conclusions: First, checkpoint detection of the primary tumor in mRCC might not provide enough information for predicting treatment effect, prognosis and making clinical decisions. Next, biopsy and resection specimens of metastases could provide more accurate evidence. In addition, the treatment effect of mRCC might be correlated with different metastatic sites. Patients with lung and/or lymph node metastases were potential candidates for immunotherapy.

## Conclusions

The expression differences between the primary and metastatic tumor of PD-1, PD-L1&2 were significant. The value of assessing immune checkpoints in the primary tumor is limited and the efficacy of immunotherapy might be associated with the site of metastasis. Therefore, histopathological evaluation of metastatic sites is worth of notice, and its accurate diagnosis might be one of the effective ways to realize the individualized treatment.

## Additional files

Additional file 1: **Table S1.** Baseline clinicopathological parameters of included patients. (DOCX 16 kb)

Additional file 2: **Table S2.** Differential expressions of PD-1,PD-L1 and PD-L2 between the primary and metastatic tumors in the paired cohort. (DOCX 16 kb)

Additional file 3: **Table S3.** Relationship between the differential expressions of PD-1, PD-L1 and PD-L2 and clinicopathological parameters in the paired cohort. (DOCX 20 kb)

Additional file 4: **Table S4.** Unvariate analysis of PFS and OS in all patients. (DOCX 19 kb)

Additional file 5: **Table S5.** Unvariate analysis of PFS and OS in paired patients. (DOCX 18 kb)

## Abbreviations

BMI: Body Mass Index
ECOG: Eastern Cooperative Oncology Group
IHC: Immunohistochemistry
IMDC: International Metastatic Renal-Cell Carcinoma Database Consortium
ISUP: International Society of Urological Pathology
mRCC: Metastatic Renal Cell Carcinoma
OS: Overall Survival
PA: Predicted Accuracy
PD-1: Programmed Death-1
PD-L1: Programmed Death-Ligand 1
PD-L2: Programmed Death-Ligand 2
PFS: Progression-Free Survival
RCC: Renal Cell Carcinoma
