# Supplementary material for: Differential expressions of PD-1, PD-L1 and PD-L2 between primary and metastatic sites in renal cell carcinoma
Source: BMC Cancer. 2019 Apr 16;19:360. doi: 10.1186/s12885-019-5578-4 (PMC6469103; doi:10.1186/s12885-019-5578-4)
Supplement: Supplementary file 1 — Table S1. Baseline clinicopathological parameters of included patients. (DOCX 16 kb) [file 12885_2019_5578_MOESM1_ESM.docx]

**Table S1. Baseline clinicopathological parameters of included patients**

|  | **Paired(N=83)** | **Metastatic(N=80)** | **P value** |
| --- | --- | --- | --- |
| **Age, n (%)** |  |  | 0.742 |
| <50 | 29(34.9) | 26(32.5) |  |
| ≥50 | 54(65.1) | 54(67.5) |  |
| **Gender, n (%)** |  |  | 0.873 |
| Male | 54(65.1) | 53(66.3) |  |
| Female | 29(34.9) | 27(33.7) |  |
| **ISUP, n (%)** |  |  | 0.01 |
| <3 | 11(15.1) | 24(33.3) |  |
| ≥3 | 62(84.9) | 48(66.6) |  |
| **Histological Type, n (%)** |  |  | 0.032 |
| ccRCC | 59(71.1) | 68(61.3) |  |
| Non-ccRCC | 24(28.9) | 12(38.7) |  |
| **Pathology, n (%)** | |  |  |
| Sarcomatoid | 6(19.4) | 2(28.6) | 0.509 |
| Necrosis | 25(80.6) | 5(71.4) | 0.491 |
| **Nephrectomy, n (%)** |  |  | 0.001 |
| Yes | 80(96.4) | 57(77.0) |  |
| No | 3(3.6) | 17(23.0) |  |
| **ECOG, n (%)** |  |  | 0.801 |
| 0-1 | 53(70.7) | 59(76.6) |  |
| ≥2 | 22(29.3) | 18(23.4) |  |
| **IMDC, n (%)** |  |  | 0.293 |
| Low | 14(18.3) | 19(28.4) |  |
| Intermediate | 43(57.3) | 37(55.2) |  |
| High | 18(24.0) | 11(16.4) |  |
| **T stage, n (%)** |  |  | 0.468 |
| <3 | 58(75.3) | 15(83.3) |  |
| ≥3 | 19(24.7) | 3(16.7) |  |
| **Metastasis, n (%)** |  |  |  |
| Lung | 6(8.1) | 17(28.8) | 0.010 |
| Brain | 7(9.5) | 12(20.3) | 0.192 |
| Liver | 1(1.4) | 2(3.4) | 0.539 |
| Bone | 18(24.3) | 22(37.3) | 0.389 |
| Lymph node | 42(56.8) | 6(10.2) | 0.001 |
| **Treatment, n (%)** |  |  |  |
| Cytokine | 14(16.9) | 8(10.0) | 0.345 |
| Target therapy | 34(41.0) | 30(37.5) | 0.882 |
| Radiotherapy | 7(8.4) | 7(8.8) | 0.673 |
| Chemotherapy | 3(3.6) | 5(6.2) | 0.360 |
| Unknown | 25(30.1) | 30(37.5) | 0.576 |
