# Supplementary material for: Differential expressions of PD-1, PD-L1 and PD-L2 between primary and metastatic sites in renal cell carcinoma
Source: BMC Cancer. 2019 Apr 16;19:360. doi: 10.1186/s12885-019-5578-4 (PMC6469103; doi:10.1186/s12885-019-5578-4)
Supplement: Supplementary file 2 — Table S2. Differential expressions of PD-1,PD-L1 and PD-L2 between the primary and metastatic tumors in the paired cohort. (DOCX 16 kb) [file 12885_2019_5578_MOESM2_ESM.docx]

**Table S2. Differential expressions of PD-1,PD-L1 and PD-L2 between the primary and metastatic tumors in the paired cohort**

|  | **Primary**  **(N=83)** |  | **Metastasis (N=83)*** | | | | | | |
| --- | --- | --- | --- | --- | --- | --- | --- | --- | --- |
|  | **n(%)** |  | **Lung/lymph node**  **n=49** | **Bone**  **n=18** | **Brain**  **n=7** | **Viscera**  **n=7** | **Adrenal gland n=2** | **Others n=5** | **Total n(%)** |
| **PD-1** |  |  |  |  |  |  |  |  |  |
| Negative | 57(68.7) |  | 16(32.7) | 11(61.1) | 5(71.4) | 5(71.4) | 1(50.0) | 4(80.0) | 43(47.8) |
| Positive | 26(31.3) |  | 33(67.3) | 7(38.9) | 2(28.6) | 2(28.6) | 1(50.0) | 1(20.0) | 47(52.2) |
| P value |  |  | 0.21 | 0.91 | 0.01 | 0.09 | NA | NA | 0.07 |
| **PD-L1** |  |  |  |  |  |  |  |  |  |
| Negative | 63(75.9) |  | 30(61.2) | 14(77.8) | 5(71.4) | 4(57.1) | 1(50.0) | 2(40.0) | 57(63.3) |
| Positive | 20(24.1) |  | 19(38.8) | 4(22.2) | 2(28.6) | 3(42.9) | 1(50.0) | 3(60.0) | 33(36.7) |
| P value |  |  | 0.08 | 0.32 | 0.43 | NA | NA | NA | 0.03 |
| **PD-L2** |  |  |  |  |  |  |  |  |  |
| Negative | 69(83.1) |  | 36(73.5) | 12(66.7) | 6(85.7) | 6(85.7) | 1(50.0) | 5(100.0) | 66(73.3) |
| Positive | 14(16.9) |  | 13(26.5) | 6(33.3) | 1(14.3) | 1(14.3) | 1(50.0) | 0(0) | 24(26.7) |
| P value |  |  | 0.95 | 0.03 | NA | NA | 0.16 | NA | 0.05 |

*7 cases have two metastatic sites. NA, not available.
