# Supplementary material for: Differential expressions of PD-1, PD-L1 and PD-L2 between primary and metastatic sites in renal cell carcinoma
Source: BMC Cancer. 2019 Apr 16;19:360. doi: 10.1186/s12885-019-5578-4 (PMC6469103; doi:10.1186/s12885-019-5578-4)
Supplement: Supplementary file 3 — Table S3. Relationship between the differential expressions of PD-1, PD-L1 and PD-L2 and clinicopathological parameters in the paired cohort. (DOCX 20 kb) [file 12885_2019_5578_MOESM3_ESM.docx]

**Table S3. Relationship between the differential expressions of PD-1, PD-L1 and PD-L2 and clinicopathological parameters in the paired cohort**

|  | **PD-1 concordance** | | **P Value** | **PD-L1 concordance** | | **P Value** | **PD-L2 concordance** | | **P Vaule** |
| --- | --- | --- | --- | --- | --- | --- | --- | --- | --- |
|  | **Yes** | **No** |  | **Yes** | **No** |  | **Yes** | **No** |  |
| **Total** | 48(57.8) | 35(47.2) |  | 56(67.5) | 27(32.5) |  | 61(73.5) | 22(26.5) |  |
| **Age, n (%)** |  |  | 0.915 |  |  | 0.831 |  |  | 0.87 |
| <50y | 17(58.6) | 12(41.4) |  | 20(69.0) | 9(31.0) |  | 21(34.4) | 8(36.4) |  |
| ≥50y | 31(57.4) | 23(42.6) |  | 36(66.7) | 18(33.3) |  | 40(65.6) | 14(63.6) |  |
| **Gender, n (%)** |  |  | 0.007 |  |  | 0.207 |  |  | 0.161 |
| Male | 37(68.5) | 17(31.5) |  | 39(72.2) | 15(27.8) |  | 37(60.7) | 17(77.3) |  |
| Female | 11(37.9) | 18(62.1) |  | 17(58.6) | 12(41.4) |  | 24(39.3) | 5(22.7) |  |
| **ISUP, n (%)** |  |  | 0.664 |  |  | 0.395 |  |  | 0.52 |
| <3 | 5(45.5) | 6(54.5) |  | 6(54.5) | 545.5) |  | 9(16.7) | 2(10.5) |  |
| ≥3 | 35(56.5) | 27(43.5) |  | 42(67.7) | 20(32.3) |  | 45(83.3) | 17(89.5) |  |
| **Histological Type, n(%)** |  |  | 0.357 |  |  | 0.35 |  |  | 0.843 |
| ccRCC | 36(61.0) | 23(39.0) |  | 38(64.4) | 21(35.6) |  | 43(70.5) | 16(72.7) |  |
| Non-ccRCC | 12(50.0) | 12(50.0) |  | 18(75.0) | 6(25.0) |  | 18(29.5) | 6(27.3) |  |
| **Pathology, n (%)** | | | | | | |  |  |  |
| Sarcoma | 2(33.3) | 4(66.7) | 0.217 | 5(83.3) | 1(16.7) | 0.379 | 3(5.0) | 3(13.6) | 0.183 |
| Necrosis | 14(56.0) | 11(44.0) | 0.924 | 20(80.0) | 5(20.0) | 0.089 | 20(33.9) | 5(22.7) | 0.225 |
| **Nephrectomy** |  |  | 0.407 |  |  | 0.199 |  |  | 0.289 |
| Yes | 45(56.3) | 35(43.7) |  | 55(68.8) | 25(31.2) |  | 58(95.1) | 22(100) |  |
| No | 3(100) | 0(0) |  | 1(33.3) | 2(66.7) |  | 3(4.9) | 0(0) |  |
| **ECOG, n (%)** |  |  | 0.698 |  |  | 0.216 |  |  | 0.565 |
| 0-1 | 35(59.3) | 24(40.7) |  | 37(62.7) | 22(37.3) |  | 44(74.6) | 15(68.2) |  |
| ≥2 | 12(54.5) | 10(45.5) |  | 17(77.3) | 5(22.7) |  | 15(25.4) | 7(31.8) |  |
| **IMDC, n (%)** |  |  | 0.137 |  |  | 0.824 |  |  | 0.52 |
| Low | 11(78.6) | 3(21.4) |  | 10(71.4) | 4(28.6) |  | 9(15.8) | 5(27.8) |  |
| Intermediate | 23(53.5) | 20(46.5) |  | 28(65.1) | 15(34.9) |  | 34(59.6) | 9(50.0) |  |
| High | 8(44.4) | 10(55.6) |  | 13(72.2) | 5(27.8) |  | 14(24.6) | 4(22.2) |  |
| **T stage, n (%)** |  |  | 0.259 |  |  | 0.122 |  |  | 0.077 |
| <3 | 36(62.1) | 22(37.9) |  | 38(65.5) | 20(34.5) |  | 40(70.2) | 18(90.0) |  |
| ≥3 | 9(47.4) | 10(52.6) |  | 16(84.2) | 3(15.8) |  | 17(29.8) | 2(10.0) |  |
| **Metastasis, n (%)** | | | | | | |  |  |  |
| Lung | 4(57.1) | 3(42.9) | 0.969 | 4(57.1) | 3(42.9) | 0.542 | 4(57.1) | 3(42.9) | 0.306 |
| Lymph node | 21(50.0) | 21(50.0) | 0.144 | 28(66.7) | 14(33.3) | 0.874 | 27(64.3) | 15(35.7) | 0.054 |
| Bone | 9(50.0) | 9(50.0) | 0.447 | 14(77.8) | 4(22.2) | 0.291 | 14(77.8) | 4(22.2) | 0.642 |
| Brain | 7(100) | 0(0) | 0.018 | 5(71.4) | 2(28.6) | 0.815 | 6(85.7) | 1(14.3) | 0.444 |
| Adrenal | 1(100) | 0(0.0) | 0.390 | 1(100) | 0(0.0) | 0.485 | 1(100) | 0(0.0) | 0.546 |
| Viscera | 4(80.0) | 1(20.0) | 0.300 | 3(50.0) | 3(50.0) | 0.343 | 4(80.0) | 1(20.0) | 0.734 |
| Others | 5(83.3) | 1(16.7) | 0.189 | 6(100) | 0(0.0) | 0.062 | 6(100) | 0(0.0) | 0.127 |
| **Treatment, n (%)** |  | |  |  |  |  |  |  |  |
| Cytokine | 8(57.1) | 6(42.9) | 0.7 | 14(100) | 0(0) | 0.003 | 11(78.6) | 3(21.4) | 0.578 |
| Targeted therapy | 18(52.9) | 16(47.1) | 0.307 | 23(67.6) | 11(32.4) | 0.629 | 23(67.6) | 11(32.4) | 0.308 |
| Radiotherapy | 4(57.1) | 3(42.9) | 0.859 | 5(71.4) | 2(28.6) | 0.682 | 6(85.7) | 1(14.3) | 0.413 |
| Chemotherapy | 2(66.7) | 1(33.3) | 0.818 | 2(66.7) | 1(33.3) | 0.9 | 2(66.7) | 1(33.7) | 0.783 |
