# Supplementary material for: Differential expressions of PD-1, PD-L1 and PD-L2 between primary and metastatic sites in renal cell carcinoma
Source: BMC Cancer. 2019 Apr 16;19:360. doi: 10.1186/s12885-019-5578-4 (PMC6469103; doi:10.1186/s12885-019-5578-4)
Supplement: Supplementary file 4 — Table S4. Unvariate analysis of PFS and OS in all patients. (DOCX 19 kb) [file 12885_2019_5578_MOESM4_ESM.docx]

**Table S4. Unvariate analysis of PFS and OS in all patients**

|  | **PFS** | | |  | **OS** | | |
| --- | --- | --- | --- | --- | --- | --- | --- |
|  | **HR** | **95% Cl** | **P value** |  | **HR** | **95% Cl** | **P value** |
| **Age** |  |  |  |  |  |  |  |
| <50 vs ≥50 | 1.078 | 0.737-1.577 | 0.699 |  | 0.950 | 0.587-1.537 | 0.834 |
| **Gender** |  |  |  |  |  |  |  |
| Male vs Female | 1.456 | 0.996-2.129 | 0.053 |  | 1.819 | 1.081-3.063 | 0.024 |
| **ISUP** |  |  |  |  |  |  |  |
| ≥3 vs <3 | 2.086 | 1.043-4.172 | 0.038 |  | 6.046 | 1.440-25.384 | 0.014 |
| **Histological type** |  |  |  |  |  |  |  |
| ccRCC vs non-ccRCC | 0.848 | 0.583-1.234 | 0.389 |  | 0.587 | 0.366-0.940 | 0.026 |
| **Sarcomatous degeneration** |  |  |  |  |  |  |  |
| Yes vs No | 1.460 | 0.628-3.393 | 0.379 |  | 1.222 | 0.373-3.999 | 0.741 |
| **Necrosis** |  |  |  |  |  |  |  |
| Yes vs No | 1.285 | 0.752-2.196 | 0.358 |  | 1.519 | 0.776-2.975 | 0.222 |
| **Nephrectomy** |  |  |  |  |  |  |  |
| Yes vs No | 0.262 | 0.145-0.474 | <0.001 |  | 0.263 | 0.134-0.514 | <0.001 |
| **ECOG** |  |  |  |  |  |  |  |
| ≥2 vs <2 | 1.849 | 1.238-2.762 | 0.003 |  | 1.911 | 1.160-3.149 | 0.011 |
| **IMDC** |  |  |  |  |  |  |  |
| Low | 1 | Ref. | Ref. |  | 1 | Ref. | Ref. |
| Median | 1.232 | 0.791-1.918 | 0.355 |  | 1.131 | 0.618-2.071 | 0.690 |
| High | 4.738 | 2.672-8.402 | <0.001 |  | 4.169 | 2.233-8.945 | <0.001 |
| **Time interval from diagnosis to metastasis** | | |  |  |  |  |  |
| Initial vs Metachronous | 2.258 | 1.501-3.397 | <0.001 |  | 3.655 | 2.214-6.032 | <0.001 |
| **T stage** |  |  |  |  |  |  |  |
| ≥3 vs <3 | 1.276 | 0.706-2.306 | 0.420 |  | 1.253 | 0.594-2.646 | 0.553 |
| **Metastasis** |  |  |  |  |  |  |  |
| Lung/LN vs others | 1.132 | 0.783-1.637 | 0.508 |  | 1.407 | 0.888-2.231 | 0.146 |
| **Individual tumor history** |  |  |  |  |  |  |  |
| Yes vs No | 0.695 | 0.321-1.504 | 0.356 |  | 0.190 | 0.026-1.371 | 0.099 |
| **Family history of tumor** |  |  |  |  |  |  |  |
| Yes vs No | 0.983 | 0.479-1.946 | 0.961 |  | 1.144 | 0.481-2.578 | 0.801 |
| **BMI(kg/m^2^)** | 0.966 | 0.891-1.048 | 0.409 |  | 0.868 | 0.780-0.965 | 0.009 |
| **Tumor size(cm)** | 0.986 | 0.945-1.029 | 0.517 |  | 1.013 | 0.967-1.061 | 0.592 |
| **Laboratory parameters** |  |  |  |  |  |  |  |
| HGB(g/L） | 0.994 | 0.982-1.006 | 0.314 |  | 0.979 | 0.964-0.994 | 0.008 |
| PLT(10^9/L） | 1.001 | 0.999-1.004 | 0.352 |  | 1.002 | 0.999-1.005 | 0.137 |
| WBC(10^9/L） | 1.122 | 1.042-1.207 | 0.002 |  | 1.148 | 1.053-1.252 | 0.002 |
| ALP(IU/L） | 1.000 | 0.996-1.004 | 0.958 |  | 1.005 | 1.001-1.009 | 0.022 |
| LDH(IU/L） | 1.001 | 0.999-1.004 | 0.255 |  | 1.003 | 1.001-1.005 | 0.013 |
| Na^1+^(mmol/L） | 1.001 | 0.999-1.002 | 0.392 |  | 1.001 | 0.999-1.002 | 0.285 |
| Ca^2+^(mmol/L) | 1.289 | 0.490-3.391 | 0.607 |  | 1.091 | 0.302-3.939 | 0.895 |
| TG(mmol/L) | 1.238 | 0.840-1.823 | 0.281 |  | 0.815 | 0.482-1.376 | 0.443 |
| CHOL(mmol/L) | 0.872 | 0.692-1.100 | 0.247 |  | 0.741 | 0.540-1.018 | 0.064 |
| HDLC(mmol/L) | 0.682 | 0.375-1.241 | 0.210 |  | 0.429 | 0.185-0.995 | 0.049 |
| LDLC(mmol/L) | 0.847 | 0.627-1.145 | 0.281 |  | 0.730 | 0.478-1.115 | 0.145 |
