# Supplementary material for: Differential expressions of PD-1, PD-L1 and PD-L2 between primary and metastatic sites in renal cell carcinoma
Source: BMC Cancer. 2019 Apr 16;19:360. doi: 10.1186/s12885-019-5578-4 (PMC6469103; doi:10.1186/s12885-019-5578-4)
Supplement: Supplementary file 5 — Table S5. Unvariate analysis of PFS and OS in paired patients. (DOCX 18 kb) [file 12885_2019_5578_MOESM5_ESM.docx]

**Table S5. Unvariate analysis of PFS and OS in paired patients**

|  | **PFS** | | |  | **OS** | | |
| --- | --- | --- | --- | --- | --- | --- | --- |
|  | **HR** | **95% Cl** | **P value** |  | **HR** | **95% Cl** | **P value** |
| **Age** |  |  |  |  |  |  |  |
| <50 vs ≥50 | 1.281 | 0.731-2.242 | 0.387 |  | 1.320 | 0.646-2.699 | 0.446 |
| **Gender** |  |  |  |  |  |  |  |
| Male vs Female | 1.243 | 0.716-2.159 | 0.440 |  | 1.026 | 0.509-2.066 | 0.943 |
| **ISUP** |  |  |  |  |  |  |  |
| ≥3 vs <3 | 2.200 | 0.910-5.318 | 0.080 |  | 7.106 | 0.961-52.544 | 0.055 |
| **Histological type** |  |  |  |  |  |  |  |
| ccRCC vs non-ccRCC | 0.879 | 0.493-1.568 | 0.662 |  | 0.685 | 0.332-1.413 | 0.305 |
| **Sarcomatous degeneration** |  |  |  |  |  |  |  |
| Yes vs No | 0.986 | 0.355-2.740 | 0.978 |  | 0.446 | 0.061-3.283 | 0.428 |
| **Necrosis** |  |  |  |  |  |  |  |
| Yes vs No | 1.204 | 0.668-2.171 | 0.537 |  | 1.592 | 0.760-3.335 | 0.218 |
| **Renal resection** |  |  |  |  |  |  |  |
| Yes vs No | 0.504 | 0.118-2.142 | 0.353 |  | 0.283 | 0.064-1.258 | 0.097 |
| **ECOG** |  |  |  |  |  |  |  |
| ≥2 vs <2 | 1.505 | 0.853-2.654 | 0.158 |  | 1.387 | 0.685-2.808 | 0.364 |
| **IMDC** |  |  |  |  |  |  |  |
| Low | 1 | Ref. | Ref. |  | 1 | Ref. | Ref. |
| Intermediate | 0.1081 | 0.536-2.178 | 0.828 |  | 1.402 | 0.510-3.854 | 0.512 |
| High | 2.377 | 1.079-5.238 | 0.032 |  | 3.765 | 1.287-11.017 | 0.016 |
| **Time from diagnosis to metastasis** | |  |  |  |  |  |  |
| Initial vs Metachronous | 0.893 | 0.529-1.508 | 0.673 |  | 2.146 | 1.067-4.314 | 0.032 |
| **T stage** |  |  |  |  |  |  |  |
| ≥3 vs <3 | 1.237 | 0.645-2.373 | 0.522 |  | 1.094 | 0.468-2.553 | 0.836 |
| **Metastasis** |  |  |  |  |  |  |  |
| Lung/lymph node vs others | 0.871 | 0.516-1.468 | 0.604 |  | 1.288 | 0.660-2.514 | 0.459 |
| **Personal history** |  |  |  |  |  |  |  |
| Yes vs No | 2.008 | 0.482-8.370 | 0.338 |  | 1.633 | 0.220-12.137 | 0.632 |
| **Family history** |  |  |  |  |  |  |  |
| Yes vs No | 1.808 | 0.550-5.945 | 0.329 |  | 0.857 | 0.205-3.587 | 0.833 |
| **BMI(kg/m^2^)** | 1.073 | 0.974-1.181 | 0.153 |  | 0.911 | 0.799-1.039 | 0.166 |
| **Tumor size(cm)** | 1.050 | 0.984-1.120 | 0.141 |  | 1.066 | 0.975-1.165 | 0.159 |
| **Laboratory results** |  |  |  |  |  |  |  |
| HGB(g/L) | 0.988 | 0.973-1.003 | 0.107 |  | 0.973 | 0.955-0.990 | 0.003 |
| PLT(10^9/L) | 1.000 | 0.997-1.003 | 0.813 |  | 1.001 | 0.998-1.004 | 0.566 |
| WBC(10^9/L) | 1.085 | 0.988-1.192 | 0.088 |  | 1.102 | 0.979-1.239 | 0.107 |
| ALP(IU/L) | 1.000 | 0.996-1.005 | 0.855 |  | 1.005 | 1.001-1.010 | 0.013 |
| LDH(IU/L) | 1.001 | 0.999-1.004 | 0.297 |  | 1.003 | 1.001-1.006 | 0.014 |
| Na^1+^(mmol/L) | 0.934 | 0.846-1.031 | 0.178 |  | 0.837 | 0.750-0.933 | 0.001 |
| Ca^2+^(mmol/L) | 1.778 | 0.447-7.073 | 0.414 |  | 3.355 | 0.368-30.604 | 0.283 |
| TG(mmol/L) | 1.327 | 0.828-2.127 | 0.240 |  | 0.949 | 0.528-1.709 | 0.863 |
| CHOL(mmol/L) | 0.907 | 0.681-1.209 | 0.506 |  | 0.846 | 0.571-1.253 | 0.404 |
| HDLC(mmol/L) | 0.717 | 0.339-1.514 | 0.383 |  | 0.536 | 0.195-1.469 | 0.225 |
| LDLC(mmol/L) | 0.832 | 0.565-1.225 | 0.351 |  | 0.756 | 0.436-1.310 | 0.756 |
